# Progressive Conduction Disease on Serial ECGs Revealing Immune Checkpoint Inhibitor–Associated Myocarditis and Myositis

**DOI:** 10.1016/j.jaccas.2026.107825

**Published:** 2026-05-20

**Authors:** Quinn M. Nelson, Jules Greenberg, Islam Abdelkarim, Joshua Lee, Carrie M. Puckett

**Affiliations:** a Department of Medicine, Oregon Health & Science University, Portland, Oregon, USA; b Oregon State University, Corvallis, Oregon, USA; c Department of Cardiology, Oregon Health & Science University, Portland, Oregon, USA; d Department of Cardiology, Portland VA Medical Center, Portland, Oregon, USA

**Keywords:** cancer, cardiac pacemaker, electrocardiogram

## Abstract

**Background:**

Immune checkpoint inhibitors are effective treatments for cancer but also carry cardiovascular toxicity risks.

**Case Summary:**

An 83-year-old male receiving cemiplimab for advanced squamous and basal cell carcinoma presented with severe back pain. One week earlier, electrocardiogram (ECG) demonstrated atrial flutter with new right bundle branch block, slowed atrial activity, and possible complete heart block with a ventricular rate of 48 beats/min, alongside rising liver enzyme abnormalities. On admission, ECG demonstrated complete heart block. Troponin and creatine kinase were elevated. He developed Society for Cardiovascular Angiography and Interventions (SCAI) Stage C cardiogenic shock treated with transvenous pacing. Progressive ophthalmoplegia, proximal weakness, and respiratory decline followed. He received high-dose methylprednisolone, intravenous immunoglobulin, and permanent pacemaker implantation. Muscle biopsy confirmed immune-mediated necrotizing inflammatory myopathy.

**Discussion:**

Serial ECG evolution suggested early immune checkpoint inhibitor myocarditis. Despite rapid immunosuppression, his disease was steroid-refractory and fatal.

**Take-Home Messages:**

Progressive conduction abnormalities on electrocardiogram may be an early manifestation of immune-checkpoint inhibitor myocarditis, before troponin elevation. Baseline and surveillance electrocardiograms can detect progressive conduction abnormalities as manifestations of early immune-checkpoint inhibitor toxicity and guide emerging mechanism-matched immunosuppression.

## Visual Summary

**Figure:**
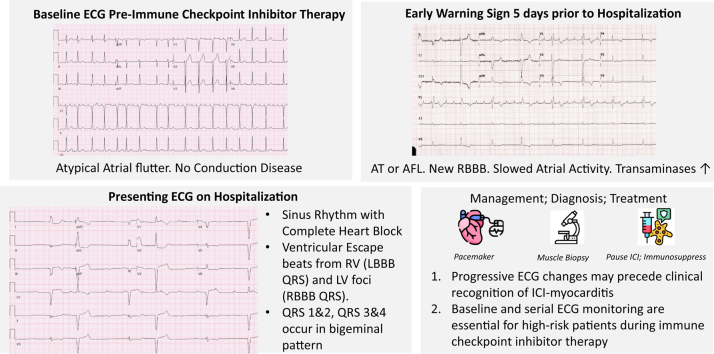

## History of Presentation

An 83-year-old male presented with 6 days of progressive stabbing back pain. He was evaluated 1 week prior at an outside hospital for similar symptoms. At that time, electrocardiogram (ECG) demonstrated atypical atrial flutter with new right bundle branch block, slowing atrial rates, and possible complete heart block with a ventricular rate of 48 beats/min. Mild elevations in transaminases were present. These findings were not attributed to immune-related toxicity. On presentation, he was alert and oriented. His exam was notable for bradycardia (HR 38) with compensated blood pressure, systolic ejection murmur, and irregular rhythm.

**Take-Home Messages**

• Progressive conduction abnormalities on electrocardiogram may be an early manifestation of immune checkpoint inhibitor myocarditis, even before troponin elevation.
• Routine, baseline, and surveillance electrocardiograms should be incorporated into immune checkpoint inhibitor–monitoring protocols for high-risk patients.
• Recent evidence highlights the potential for mechanism-matched immunosuppression.

## Past Medical History

The patient presented with a history of paroxysmal atrial fibrillation/flutter (CHA_2_DS_2_-VASc of 4) on apixaban, chronic obstructive pulmonary disease, abdominal aortic aneurysm, and advanced basal cell and squamous cell carcinoma having received only 1 dose of cemiplimab.

**Visual Summary:**
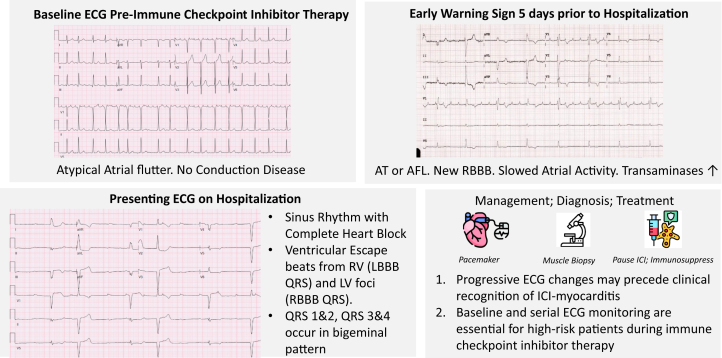
Progressive Conduction Disease on Serial ECGs Revealing Immune Checkpoint Inhibitor-Associated Myocarditis and Myositis Baseline ECG pre-immune checkpoint inhibitor, 1 year prior to hospitalization was notable for atypical atrial flutter (atrial rate of ∼300 bpm) and variable block (positive P wave in V1 and lead III) with ventricular rate of 102. The patient then presented 5 days prior to hospitalization with an ECG showing atrial tachycardia or atypical atrial flutter (atrial rate is slow with rate of 180-200 bpm and no distinguishable P wave in other leads) with variable conduction at the beginning of tracing, then fixed QRS intervals: V1; QRS (qR, RBBB) and buried P within QRS. At this time the patient also developed elevated transaminases. The patient ultimately presented to the hospital with severe back pain and ECG on admission was notable for sinus rhythm with complete heart block and ventricular escape beats from right ventricular (LBBB QRS) and left ventricular foci (RBBB QRS); marching out all of the P waves, none of the QRS complexes are associated with consistent PR intervals; suggesting against alternating bundle branch block or phase 4 block (Note that QRS 1 & 2, QRS 3 & 4) seem to occur in a bigeminal pattern. This patient was treated with transvenous pacemaker for complete heart block complicated by Society for Cardiovascular Angiography & Interventions Stage C Cardiogenic shock. The patient developed progressive ophthalmoplegia, proximal muscle weakness, and respiratory decline. He was treated with high-dose methylprednisolone, intravenous immunoglobulin, and permanent pacemaker implantation. Muscle biopsy confirmed immune-mediated necrotizing inflammatory myopathy. Despite rapid immunosuppression, his disease was steroid-refractory and fatal. Pacemaker icon made by Freepik from www.flaticon.com CC BY 3.0. Microscope icon by Smalllike from Noun Project CC BY 3.0. Immunotherapy icon by Kliwir Art from www.flaticon.com CC BY 3.0.

## Differential Diagnosis

The chest pain, ECG changes, and elevated cardiac enzymes presented a differential diagnosis that included acute coronary syndrome, complete heart block, myositis with secondary cardiac involvement, cardiogenic shock complicated by multiorgan failure, sepsis-related myocardial injury, and immune checkpoint inhibitor (ICI)-associated myocarditis and myopathy.

## Investigations

Baseline ECG 1 year prior to ICI therapy revealed atypical atrial flutter (Figure 1). ECG obtained 5 days prior to hospitalization demonstrated atypical atrial flutter with variable conduction at the beginning of the tracing, then fixed QRS intervals with right bundle branch block (Figure 2). Upon admission to the hospital, the patient's ECG was notable for sinus rhythm (rate of 88 beats/min) with complete heart block and ventricular escape beats from right ventricular (LBBB QRS) and left ventricular foci (RBBB QRS) (Figure 3). Notable lab studies on admission revealed a leukocytosis at 15.4, elevated transaminases (AST 661 U/L, ALT 502 U/L), elevated alkaline phosphatase (152 U/L), elevated lactate at 3.3 (mmol/L), elevated high-sensitivity troponin T at 1,716 (ng/mL), elevated creatine kinase MB at 179 (ng/mL), and elevated creatine phosphokinase at 3,888 (U/L). Invasive coronary angiography demonstrated normal coronary arteries without obstructive disease. On admission, transthoracic echocardiography after temporary pacemaker placement revealed preserved left ventricular systolic function (ejection fraction 55–60%) and a severely dilated left atrium. The patient was in a paced rhythm at 60 beats/min, with a cardiac output of 3.37 L/min estimated by left ventricular outflow tract velocity–time integral. Stroke volume was calculated at 56.2 mL, with a dimensionless index of 0.68 and a cardiac index of 1.94 L/min/m^2^; FDG cardiac PET was performed in lieu of cardiac MRI due to pacemaker placement and demonstrated no abnormal myocardial FDG uptake. Deltoid muscle biopsy revealed severe active inflammatory necrotizing myopathy with clusters of myofiber necrosis associated with endomysial lymphocyte and macrophage-rich inflammation, findings consistent with an immune-mediated necrotizing/inflammatory myopathy secondary to ICI therapy.

**Figure 1:**
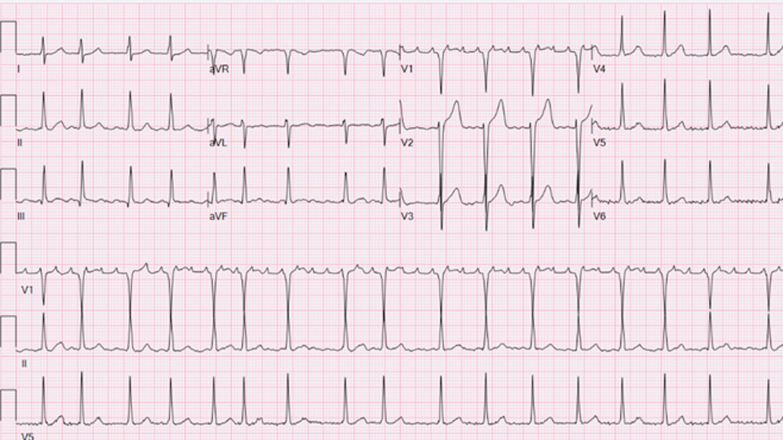
Baseline Electrocardiogram Pre-Immune Checkpoint Inhibitor, 1 Year Prior to Hospitalization (i) Atypical atrial flutter (atrial rate of ∼300 beats/min) with variable block (positive P wave in V1 and lead III) with ventricular rate of 102.

**Figure 2:**
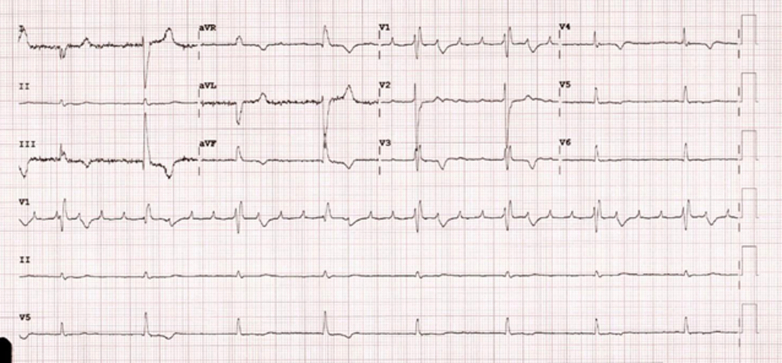
Electrocardiogram 1 Week Prior to Hospitalization and 2 Months After Immune Checkpoint Inhibitor Initiation (i) Atrial tachycardia or atypical atrial flutter (atrial rate is slow with rate of 180-200 beats/min and no distinguishable P-wave in other leads) with variable conduction at the beginning of tracing, then fixed QRS intervals. V_1_: QRS (qR, RBBB) and buried P within QRS.

**Figure 3:**
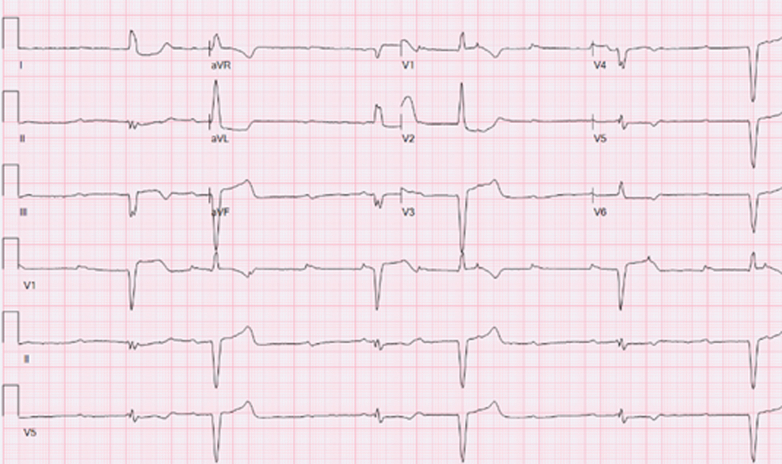
Electrocardiogram Upon Admission to the Hospital (i) Sinus rhythm (rate of 88 beats/min) with complete heart block and ventricular escape beats from right ventricular (LBBB QRS) and left ventricular foci (RBBB QRS). Marching out all of the P waves, none of QRS complexes are associated with consistent PR intervals, suggesting against alternating bundle branch block or phase 4 block. Note that QRS 1&2 and QRS 3&4 seem to occur in a bigeminal pattern.

## Management

The patient was managed with urgent temporary active fixation pacemaker which was then upgraded to a permanent pacemaker. The ICI was stopped. High-dose steroids with methylprednisolone 1 mg/kg daily was started. Days later, intravenous immunoglobulin was started at 0.5 g/kg daily. Serial negative inspiratory force was monitored. The patient received prompt and immediate care in the cardiac intensive care unit. Cardiac biopsy was deemed too high risk. Multidisciplinary care was obtained with rheumatology, oncology, cardiology, and critical care teams.

## Outcome and Follow-up

Despite prompt immunosuppressive therapy, the patient developed worsening neuromuscular weakness, respiratory compromise, and died.

## Discussion

ICI-associated myocarditis-myositis-myasthenia gravis (MMM) syndrome is a rare condition that is associated with therapies targeting the Programmed Cell Death Protein 1 (PD-1)/Programmed Cell Death Ligand 1 (PD-L1) and Cytotoxic T-Lymphocyte-Associated Protein 4 (CTLA-4) pathways.^1^, ^2^, ^3^ Conduction abnormalities are among the most common presenting features and can precede ventricular dysfunction and biochemical evidence of myocarditis.^3^^,^^4^

Current medical literature contains heterogenous recommendations for baseline ECG and troponin assessments prior to ICI initiation with some suggesting serial monitoring in high-risk patients.^4^, ^5^, ^6^, ^7^, ^8^ High-risk patients include those with dual ICI (anti-PD-1/PDL-1 + anti-CTLA-4) therapy, early treatment cycles (first 1-3 doses), cardiovascular disease, melanoma, thymic epithelial tumors, autoimmune disease, myasthenia gravis, age (>75 years old), and antiacetylcholine receptor autoantibodies.^3^^,^^7^^,^^8^

Once recognized, MMM syndrome is frequently fatal; however, recent case reports and review articles have highlighted important mechanistic distinctions among specific ICI's and corresponding therapeutic implications. CTLA-4 blockade amplifies early T‑cell priming by disinhibiting costimulatory signaling at the level of antigen presentation, whereas PD‑1/PD‑L1 inhibition predominantly augments peripheral effector T‑cell activation and interferon‑driven inflammatory cascades. Accordingly, “mechanism-matched” immunosuppression for ICI-associated MMM syndrome has the theoretical potential to more effectively reverse immune toxicity than glucocorticoids alone.^5^^,^^7^, ^8^, ^9^ New evidence supports CTLA-4 agonism (abatacept) for CTLA-4-driven disease and Janus Kinase (JAK) - Signal Transducer and Activator of Transcription (STAT) pathway inhibition (ruxolitinib) or cytokine blockage for PD-1-mediated, steroid refractory inflammation.^8^

In our case, serial prehospitalization ECGs demonstrated progression of early conduction disease concurrent with biochemical evidence of myositis, representing a missed opportunity for early recognition of immune-related myocarditis and potential immunosuppressive intervention. The patient had postponed oncology follow-up and declined his second infusion, further limiting structured monitoring. Notably, abatacept is currently being studied in a randomized control trial for treatment of ICI myocarditis (ATRIUM Trial; NCT05335928).

## Conclusions

Serial ECGs demonstrating evolving conduction abnormalities provided a critical early diagnostic clue to ICI-associated myocarditis. Prompt and early recognition has the potential for timely immunosuppression and improved outcomes: mitigating the catastrophic multisystem immune toxicity. Recent evidence suggests that mechanism-matched immunosuppression should be used for severe and steroid-refractory cases. The increasing use of ICIs with mixed guidance on cardiotoxicity screening reinforces the need for prospective research to develop both preventative and treatment strategies.

## Funding Support and Author Disclosures

This case was reviewed with the local VA Health System Privacy Officer and granted permission for publication. The authors have reported that they have no relationships relevant to the contents of this paper to disclose.

## References

[1] Lyon A.R., Yousaf N., Battisti N.M.L., Moslehi J., Larkin J. (2018). Immune checkpoint inhibitors and cardiovascular toxicity. Lancet Oncol.

[2] Mahmood S.S., Fradley M.G., Cohen J.V. (2018). Myocarditis in patients treated with immune checkpoint inhibitors. J Am Coll Cardiol.

[3] Escudier M., Cautela J., Malissen N. (2017). Clinical features, management, and outcomes of immune checkpoint inhibitor–related cardiotoxicity. Circulation.

[4] Raschi E., Rossi S., De Giglio A. (2023). Cardiovascular toxicity of immune checkpoint inhibitors: a guide for clinicians. Drug Saf.

[5] Brahmer J.R., Lacchetti C., Schneider B.J. (2018). Management of immune-related adverse events in patients treated with immune checkpoint inhibitor therapy: American Society of Clinical Oncology Clinical Practice Guideline. J Clin Oncol.

[6] Schneider B.J., Naidoo J., Santomasso B.D. (2021). Management of immune-related adverse events in patients treated with immune Checkpoint Inhibitor Therapy: ASCO Guideline Update. J Clin Oncol.

[7] Lyon A.R., López-Fernández T., Couch L.S. (2022). 2022 ESC Guidelines on cardio-oncology developed in collaboration with the European Hematology Association (EHA), the European Society for Therapeutic Radiology and Oncology (ESTRO) and the International Cardio-Oncology Society (IC-OS). Eur Heart J.

[8] Herrmann J., Barac A., Carver J. (2026). Immune checkpoint inhibitor–associated cardiovascular toxic effects. JAMA Oncol.

[9] Halloran S., Patel R., Tilkens B. (2025). Myocarditis, myositis, and myasthenia gravis. JACC Case Rep.

